# Evaluation of the Antimicrobial Activity of Four Plant Essential Oils against Some Food and Phytopathogens Isolated from Processed Meat Products in Egypt

**DOI:** 10.3390/foods11081159

**Published:** 2022-04-16

**Authors:** Shahenda S. Elshafie, Hazem S. Elshafie, Rasha M. El Bayomi, Ippolito Camele, Alaa Eldin M. A. Morshdy

**Affiliations:** 1 Food Control Department, Faculty of Veterinary Medicine, Zagazig University, Zagazig 44519, Egypt; dr_shahy.salah@yahoo.com (S.S.E.); rmazab_2010@yahoo.com (R.M.E.B.); ammorshdy@vet.zu.edu.eg (A.E.M.A.M.); 2 School of Agricultural, Forestry, Food and Environmental Sciences, University of Basilicata, 85100 Potenza, Italy; ippolito.camele@unibas.it

**Keywords:** aromatic plants, natural products, food contamination, food preservation, sensory test, molecular identification

## Abstract

Synthetic preservatives are widely utilized by the food industry to inhibit the microbial contamination and increase food safety and shelf life. The excessive utilization of synthetic preservatives can have a negative impact on human health and the environment. There is a great interest to find out natural substances as possible food-preservatives. The consumers’ preference for food products with natural ingredients prompted food manufacturers to utilize natural-based preservatives in their production. It is worth noting that plant essential oils (EOs) among the natural-based substances have been efficiently used as antimicrobial agents against phyto- and food pathogens. The current study was conducted to evaluate the microbial contamination of three industrial meat products from five governorates in Egypt, identify the predominant bacterial and fungal isolates and determine the antimicrobial efficacy of some EOs (thyme, fennel, anise and marjoram) against the most predominant microbial isolates. A sensory test was also performed to estimate the customer preferences for specific organoleptic aspects of meat products after EOs treatment. Results showed that there is a promising antimicrobial activity of all studied EOs against some microbial isolates in a dose-dependent manner. In particular, thyme EO showed the highest significant antibacterial activity against *P.* *fluorescence* and *E. coli.* Whereas the marjoram EO showed the highest activity against *P. aeruginosa*. In addition, the sensory test revealed that the treatment with anise and marjoram EOs showed the highest acceptability by the testers and did not show significant differences on the organoleptic properties with respect to control. As overall, the obtained results of the current research are promising and proved feasibility of employing plant EOs as possible preservatives for processed meat products.

## 1. Introduction

Meat is the flesh of animals, sheep, cattle, goat and swine, which serves as food [1]. The huge increase in world population has led to increasing the production of industrial meat products as popular common foods and their world consumption has increased recently [2]. The preparation process may face the risk of microbial contamination during the different phases of production. In particular, Egypt is among the countries with the highest consumption of industrial meat products, either due to high population or the traditional food aptitude [3]. Among the most popular meat products in Egypt and the Middle East area are sausage, minced meat, luncheon meat, hot dog, burger and basterma.

Meat products such as basterma, luncheon meat, burger, minced meat, sausage and hot dog are types of meat that have high nutritive value and also preferred by consumers for their attractive taste and low cost in comparison to raw meat. Therefore, the meat industry has increased recently and several meat products have become an important part of the daily diet of consumers in the Middle East and developing countries. They are also the preferred products of young people and children.

Consumers are worried not only about the taste of meat, but also about its nutrition, safety and wholesomeness [4]. On the other hand, consumers are more careful not only about the nutritional properties of the consumed meat but also about the sanitation level.

For this reason, many researchers have reported that the microbial hazards associated with processing of many meat products may be strongly correlated to the processing water, processing slabs, utensils, spices and raw meat itself [5]. On the other hand, food spoilage is a serious widely neglected problem, mainly because of mycotoxins producing fungi due to poor harvesting practices, inappropriate drying, handling, packaging, storage and transport conditions [6,7].

Several pieces of research have been conducted on the evaluation of the microbial quality of industrial meat products, and revealed the presence of some mycotoxins, fungal secondary metabolites, produced particularly from some strains of different species of *Penicillium*, *Aspergillus* and *Fusarium* [1,8,9]. These metabolites, which can be immunosuppressive, mutagenic, carcinogenic, genotoxic and/or teratogenic agents, are toxic for animals and humans, even at very low concentrations [10,11]. Furthermore, mycotoxins can have adverse health effects either acute and/or chronic according to the ingested quantity [12]. Furthermore, some researchers have reported that bacterial contamination of meat products might be related to common bacterial food pathogens such as *Bacillus cereus*, *Pseudomonas aeruginosa*, *P. fluorescens*, *Staphylococcus aureus* and *Salmonella* species [13,14,15,16].

Excessive usage of synthetic chemicals for controlling human and food pathogens and phytopathogens has increased microbe resistance to antibiotics and pesticides, as well as caused detrimental consequences for the environment and human health [17,18]. Therefore, the search for new eco-friendly alternatives for controlling food and phytopathogens became necessary [19,20]. The discovery of natural antimicrobial substances can be useful for combating foodborne pathogens, decreasing microbial resistance, minimizing environmental risk and extending the shelf life of food products [21,22].

Plant essential oils (PEOs) have been used for centuries in different cultures and communities around the world for different purposes, such as healing agents and for domestic uses [23,24,25]. Recently the PEOs were considered important natural alternatives to chemical pesticides and food preservatives due to their potential antimicrobial effects [26,27,28]. In particular, several common aromatic PEOs belonging to the *Lamiaceae*, *Apiaceae*, *Rutaceae* and *Verbanaceae* families commonly grew in the Mediterranean region and showed promising antimicrobial effects against several human and food pathogens and phytopathogens [29]. Although the potential microbicidal effect of many PEOs as well as their single constituents is acknowledged, the selection of the most suitable EO and the determination of the best efficient dose should be completed to avoid any possible side effects [29].

The main aim of this study is to evaluate the antimicrobial activity of four essential oils extracted from the aerial parts of *Thymus vulgaris* L. (thyme), *Foeniculum vulgare* L. (fennel), *Pimpinella anisum* L. (anise) and *Majorana hortensis* L. (marjoram) against nine food pathogens isolated from three studied industrial meat products (minced meat, sausage, burger) from Egypt. In addition, a sensorial test was carried out for evaluation of some organoleptic properties (color, odor, consistency, taste) of the studied treated meat products with the four tested essential oils.

## 2. Materials and Methods

### 2.1. Sampling and Preparation

Twenty-five samples from each studied beef meat product (minced meat, sausage and burger) were randomly collected from five different meat stores in Egypt: Cairo (CA), Qalubia (QA), Giza (GZ), Behira (BH) and Sharkia (SH) with different levels of hygiene, sterilization and quality in the period between February and May 2021. The samples were purchased aseptically in sterile polyethylene bags and immediately transported to the laboratory for bacteriological investigation in an ice box.

Twenty-five grams of each sample were aseptically put into a sterile blender containing 225 mL of 0.1 percent sterile buffered peptone water (BPW, HiMedia, M614-500G) under complete aseptic conditions and blended at room temperature (25 °C). One mL of the homogenate was transferred to a sterile test tube containing 9 mL of 0.1 percent BPW, and then tenfold serial decimal dilutions up to 10^−6^. The tested dilutions for microbial counting assays have been selected according to the regulations of the Egyptian Organization for Standards & Quality (2005).

### 2.2. Determination of Microbial Contamination

#### 2.2.1. Total Bacterial Count

One mL of previously prepared dilutions 10^−5^ and 10^−6^ in the case of the minced meat and sausage, and 10^−2^ and 10^−3^ in the case of the burger were placed in a sterile Petri dish and about 15 mL of plate count agar media (Liofilchem, Roseto degli Abruzzi, Italy) was poured into the dish, and then incubated at 37 °C for 24 h (pouring method), according to ISO 4833-1:2013 (E) [30]. The suspected colonies (pin head, creamy white) have been counted and calculated per each gram of sample with five replicates.

#### 2.2.2. Total Enterobacteriaceae Count

The total *Enterobacteriaceae* count was carried out according to ISO 21528-2:2004 (E) [31]. One hundred µL from the dilutions (10^−1^ and 10^−2^) was distributed on violet-red bile glaucous (VRBG, HiMedia, M581BP) petri dishes and then incubated at 37 °C for 24 h. The suspected colonies (large purple to red) surrounded by purple halo were counted and calculated per each gram of sample with five replicates.

#### 2.2.3. Total Pseudomonas Count

The isolation and total count of *Pseudomonas* spp. was carried out in Petri dishes containing approximately 15 mL of *Pseudomonas* Selective Agar (CFC) medium. The CFC is the selective medium for the isolation and enumeration of *Pseudomonas* spp. from meat and meat products according to ISO 13720:2010 (E) [32]. Briefly, for the isolation, 1 mL of the higher dilution dilutions (10^−2^ and 10^−3^) was spread on CFC plates and incubated at 25 °C for 48 h. After that, the plates containing less than 150 colonies were considered for counting and calculated per each gram of sample with five replicates. Successively, five colonies were picked randomly and recultured on CFC for molecular identification of the species. 

#### 2.2.4. Detection of *Escherichia coli*

*Pre enrichment for stock preparation*. Twenty-five g of each meat sample was mixed with 225 mL of sterile BPW to make a dilution of 1:10 and incubated at 37 °C for 24 h as stock solution. 

*Enrichment*. One mL of stock solution was added to nine mL of Lauryl Sulphate Tryptose broth (LST), the selective medium for detection and enumeration of *Escherichia coli* according to ISO 4831:2006 (E) [33] and ISO 7251:2005 (E) [34], and incubated at 37 °C for 48 h. A loopful of the above LST broth was streaked on Eosin Methylene Blue agar (EMBA, HiMedia 22–500 g) and incubated at 37 °C for 24 h.

The agar plates were examined for growth of *E. coli*, where the colonies appeared as typical green-metallic on EMBA. Single colonies were inoculated onto Nutrient Agar (NA, HiMedia) and incubated at 37 °C for 24 h for further identification by using the biochemical assays (Bergey’s Manual of Determinative Bacteriology).

#### 2.2.5. Total Yeast and Mold Count

The isolation and total count of mold was carried out in Petri dishes containing 15 mL of Sabouraud Dextrose Agar (SDA, HiMedia M063–500 g) media supplemented with chloramphenicol (broad spectrum antibiotic). One hundred µL of the higher dilution (10^−2^) was inoculated into SDA plates and incubated at 25 °C for 5–7 days according to ISO 21527-1:2008 (E) [35].

### 2.3. Extraction of Essential Oils

The studied essential oils (EOs) were extracted from the aerial parts of thyme, fennel, anise and marjoram cultivated in the greenhouse of the School of Agricultural, Forestry, Food and Environmental Sciences (University of Basilicata, Potenza—Italy) in the period from February 2021 to July 2021.

Briefly, the aerial parts of the studied plants were dried in the oven at 65 °C for 48 h, then, one hundred grams were ground in a Laboratory Blender (Waring, Merck KGaA, Darmstadt, Germany) and subjected to hydrodistillation for 3 h using a Clevenger-type apparatus, according to the standard procedure described in the European Pharmacopoeia [36]. The distilled EOs were solubilized in *n*-hexane, filtered through anhydrous Na_2_SO_4_ and stored under N_2_ at 4°C in darkness for subsequent analyses. The analysis of chemical composition of the studied EOs was previously carried out using GC-MS and the detailed information about their main constituents was reported in the discussion section.

### 2.4. Antimicrobial Activity

#### 2.4.1. Bacterial Isolation and Identification

The most abundant bacterial isolates were recultured on King B (KB) media [37] and Luria Burtani’s (LB) media [38] and incubated after that at 30 ± 2 °C for 72 h for obtaining the pure cultures. The obtained pure cultures were successively identified by the molecular method and used for the antibacterial activity assay.

For identification, the total genomic nucleic acids (gDNA) from pure bacterial isolates were carried out using DNeasy Plant Mini Kit (Qiagen, Heidelberg, Germany), according to the manufacturer’s instructions. The gDNA was amplified based on polymerase chain reaction (PCR) using the universal primer pair Y1/Y2 of the ribosomal DNA [39]. The obtained amplicons were directly sequenced and compared with those available in the GenBank nucleotide archive using the Basic Local Alignment Search Tool software BLAST (Bethesda, Rockville Pike, MD, USA) [40].

#### 2.4.2. Mold Isolation and Identification

The most abundant fungal isolates were recultured on Potato Dextrose Agar media (PDA) and incubated at 22 ± 2 °C for 96 h to obtain the pure cultures. The obtained pure cultures were successively identified by morphological and molecular methods and used for the antifungal activity assay.

The pure cultures were firstly identified based on their morphological features and examined by light microscope (Axioskop—ZEISS, Oberkochen, Germany). The exact identification was confirmed using the molecular method based on polymerase chain reaction (PCR) [41]. The gDNAs were extracted from each pure fungal isolate using DNeasy Plant Mini Kit (Qiagen, Heidelberg, Germany), according to the manufacturer’s instructions. The gDNAs were amplified using the universal primer pair ITS4/ITS5 of the ribosomal DNA [42]. The obtained amplicons were directly sequenced and the resulting sequences were compared with those available in the GenBank nucleotide archive using the Basic Local Alignment Search Tool software BLAST (Bethesda, Rockville Pike, MD, USA) [40].

#### 2.4.3. In Vitro Antibacterial Assay

The disc diffusion method was carried out for evaluating the antibacterial activity of the studied EOs against the bacterial isolates [43,44]. A suspension of each bacterial isolate was prepared in sterile distilled water and incorporated in soft agar (0.7% at 9:1 *v*/*v*). The prepared suspension was adjusted at 10^8^ colony form unit (CFU)/mL (corresponding to 0.2 nm optical density—OD) using Turbidimetry (Biolog, USA). Four mL of each bacterial suspension was poured into a Petri dish (Ø 90 mm) containing 10 mL KB media and left for 10 min under laminar flow. Blank Discs (Ø 6mm) (OXOID, Milan, Italy) were placed on KB-plate surfaces and 15 µL from each EO at concentrations of 80, 40, 20, 2.0 and 1.0 mg/mL, prepared as emulsion with 0.2% Tween, were applied over the disks. Tetracycline at 0.16 mg/mL was used as positive control (C+ve) based on the medical and veterinary rules. All plates were incubated at 37 °C for 24 h and the bactericidal activity was evaluated by measuring the diameter of inhibition zones (mm), and the growth inhibition percentage (GIP) was calculated following the Equation (1). All tested treatments were carried out in triplicates and the standard deviations (SDs) were also calculated.

(1) $$\mathrm{GIP}\;\left(\%\right)=\frac{\mathrm{Gc}-\mathrm{Gt}}{\mathrm{Gc}}\times 100$$

where: GIP represents the bacterial growth inhibition percentage; Gc: average diameter of bacterial grown in C-ve plates (mm); Gt: average diameter of inhibition zone in EO-treated plates (mm).

#### 2.4.4. In Vitro Antifungal Assay

For antifungal activity assay, the studied plant EOs were tested at the following concentrations 2.0, 1.0 and 0.5 mg/mL incorporated directly into PDA media [45,46]. Fourteen mL of the PDA-aliquots supplemented with each concentration were poured into Petri dishes (Ø 90 mm). Fungal disks (Ø 5 mm) of fresh cultures (96 h) were individually inoculated in the center of each Petri dish and incubated at 22 ± 2 °C for 120 h under darkness. Petri dishes containing only PDA were inoculated with each fungal isolate as negative control (C-ve). The diameter of mycelium growth was measured in mm ±SDs of three replicates [47,48,49] and the growth inhibition percentage (GIP) was calculated using the Zygadlo Equation (2) [50] compared to cycloheximide (0.1 mg/mL) as C+ve:

(2) $$\mathrm{GIP}\;\left(\%\right)=100-\left[\frac{\mathrm{Gc}-\mathrm{Gt}}{\mathrm{Gc}}\;\times \;100\right]$$

where: GIP represents the fungal growth inhibition percentage; Gc: average diameter of mycelium in C-ve plates (mm); Gt: average diameter of mycelium in EO-treated plates (mm). 

### 2.5. Sensory Test of Organoleptic Properties

A sensory test was performed for evaluating the effect of the EOs treatments on some organoleptic properties of the minced meat, the basic constituent of the three studied meat products. A semi-structured questionnaire consisting of four questions was administered to five women and five men, regular consumers from University of Basilicata, (Potenza, Italy) and their ages ranged between 28 to 65 years. The following organoleptic properties: odour, consistency, color and tasting were evaluated by the testers compared to the control sample (non-treated meat) using the scale as illustrated in Table 1. The used concentrations for EOs (thyme 40, fennel 80, anise 60 and marjoram 60 mg/mL) were the lowest which achieved the microbial inhibition obtained from the in vitro antimicrobial assay. The treatment was carried out using 1.4 mL from each EO added to 140 g of meat. The quantity of EO treatment (1.4 mL/140 g) was the same as normally used in the industrial meat products’ company in Cairo (Egypt) for treating the meat with sulfuric acid as a preserving substance (10 L/1 Ton). The test was performed for each tester in the same environment and conditions in order to guarantee equivalence measurements and the collection phase was carried out simultaneously. On the other hand, the pH value for each treated sample was also measured by dissolving 1 g of meat in 10 mL of Buffered Peptone Water (BPW, HiMedia), shaken for 30 min using Orbital Shaker (Merck KGaA, Darmstadt, Germany) and then measured by Microprocessor pH meter (Hanna, Merck KGaA, Darmstadt, Germany).

### 2.6. Statistical Analysis

The obtained results were subjected to one-way ANOVA for the statistical analysis. The significance level of the results was checked by applying Tukey B Post Hoc multiple comparison test with a probability of *p* < 0.05 using the statistical Package for the Social Sciences (SPSS) version 13.0 (Prentice Hall: Chicago, IL, USA, 2004).

## 3. Results

### 3.1. Microbial Contamination

#### 3.1.1. Isolation and Total Counting

Results of the microbiological analysis of the minced meat from five different governorates in Egypt are illustrated in Table 2. In particular, the total count of aerobic bacteria was the highest in QA, followed by the GZ governorate, whereas the samples from the BH, SH and CA governorates showed the lowest count of bacterial contamination. In addition, the highest occurrence of *Pseudomonas* count was found in the QA sample, whereas the lowest count was observed in the CA and GZ samples (Table 2). The highest count of *Enterobacteriaceae* and *E. coli* was detected in the QA and GZ samples, while moderate amounts were found in the CA, SH and BH samples (Table 2). On the other hand, the greatest levels of mold and yeast contamination were found in the case of the CA and BH samples, while the lowest value was detected in the SH and QA samples (Table 2).

Regarding the microbiological analysis of the sausage product, the obtained results showed that the sample of SH had the highest TCB, whereas the lowest level was observed in GZ (Table 3). In terms of *Pseudomonas*, the highest value was observed in the samples of QA and CA, while sample from GZ, SH and BH had the lowest rate (Table 3). In addition, the QA sample had the greatest level of *Enterobacteriaceae* and *E. coli*, but BH had the lowest value (Table 3). Regarding the mold and yeast, the sample from SH demonstrated the highest percentage followed by QA and BH (Table 3).

The results of the microbiological analysis of the burger product are illustrated in Table 4. In particular, the samples from QA had the highest total counting bacterial contamination, whereas, SH sample had the lowest value (Table 4). There are considerable numbers of *Pseudomonas* in CA slightly higher than and QA, respectively, whereas the samples from GZ showed the lowest rate (Table 4). In terms of *Enterobacteriaceae* and *E. coli*, the samples from CA and SH had the greatest value, while the samples from BH had the lowest rate (Table 4). Regarding the mold and yeast contamination, the samples from CA and SH demonstrated moderately contamination, whilst BH had shown the highest value (Table 4).

#### 3.1.2. Bacterial Molecular Identification

The PCR amplification for the three most abundant bacterial isolates from the studied meat samples produced amplicons with molecular weights of approximately 430 bp. No amplification was observed in the case of the negative control. The obtained amplicons were directly sequenced (BMR Genomics, Padova, Italy) and the obtained sequences were compared with those available in the GenBank nucleotide archive using the Basic Local Alignment Search Tool software BLAST (Bethesda, Rockville Pike, MD, USA) [40]. The sequences analysis for the bacterial isolates showed a high similarity with the sequences already presented in the GenBank BLAST database as follows: 99.50% for *Pseudomonas fluorescence*, 98.80% for *P. aeruginosa* and 99.30% for *Escherichia coli*.

#### 3.1.3. Mold Molecular Identification

The PCR amplification of the six most abundant mold isolates from the studied meat samples produced amplicons with molecular weights of approximately 550 bp. No amplification was observed in the case of the negative control. The obtained amplicons were directly sequenced (BMR Genomics, Padova, Italy) and the obtained sequences were compared with those available in the GenBank nucleotide archive using the Basic Local Alignment Search Tool software (BLAST—USA) [40]. The sequences analysis for each isolate showed high similarity with the sequences already presented in the GenBank database as follows: 99.2% for *Aspergillus flavus*, 99.47% for *A. niger*, 98% for *Penicillium digitatum*, 99% for *P. expansum*, 99.83% for *P. commune* and 99.82% for *P. italicum*, which confirmed the morphological identification.

### 3.2. In Vitro Antimicrobial Activity of EOs

#### 3.2.1. Antibacterial Activity Assay

**Figure 1:**
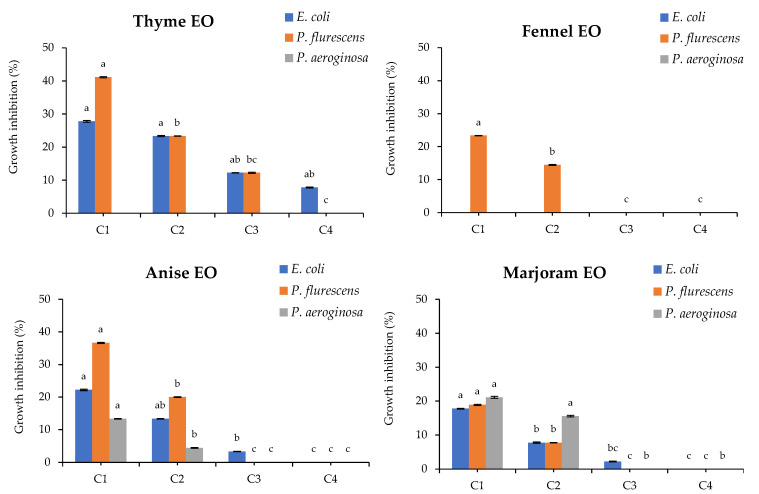
Antibacterial activity of the studied essential oils against 3 isolated bacteria at 4 different concentrations. Where: C1, C2, C3 and C4 are the tested concentrations of each studied EO at 80, 60, 40 and 20 mg/mL, respectively. Bars with different letters are significant according to Waller–Duncan multiple comparison test post hoc test at *p* < 0.05 using SPSS statistical analysis software.

The antibacterial activity of the studied EOs against the bacterial isolates was illustrated in Figure 1. In particular, the thyme EO showed the highest significant antibacterial activity against *P.* *fluorescence* and *E. coli* where the growth inhibition percentages were 41.2 and 27.7%, respectively, using the highest concentration. The same EO did not show any activity against *P. aeruginosa* (Figure 1). Fennel EO showed a moderate activity against *P.* *fluorescence* at the two higher concentrations (80 and 60 mg/mL) (Figure 1). On the other hand, anise and marjoram EOs were able to inhibit all tested bacterial strains at the two higher concentrations (80 and 60 mg/mL). Furthermore, anise EO explicated the maximum activity against *P.* *fluorescence* only at 80 mg/mL. Marjoram EO showed activity against *P. aeruginosa* even at 60 mg/mL, not significantly different from the highest tested concentration (80 mg/mL), whereas anise EO showed moderate activity against the same bacterium at 80 mg/mL.

#### 3.2.2. Antifungal Activity Assay

**Figure 2:**
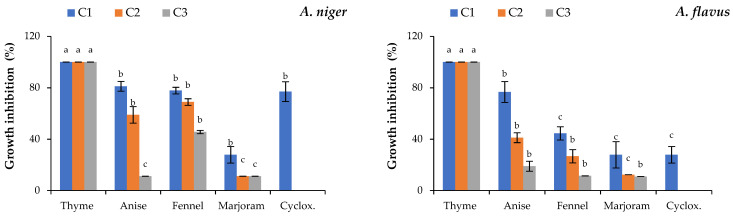

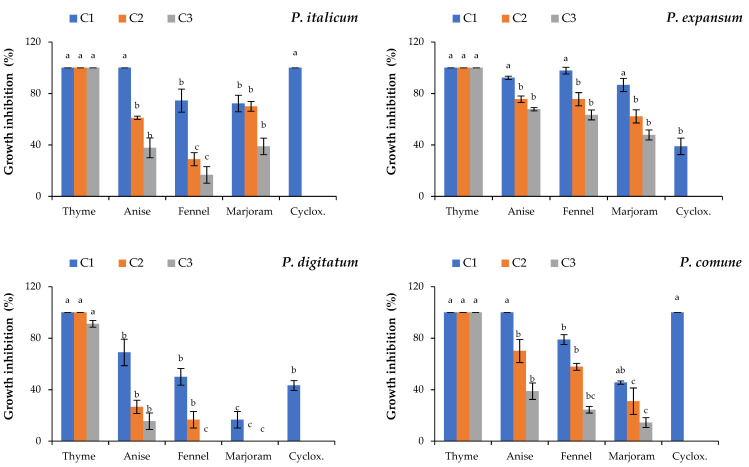
Antifungal activity of the studied essential oils against the isolated fungi at three different concentrations. Where: the tested concentrations C1, C2 and C3 are of each studied EO at 2.0, 1.0 and 0.5 mg/mL, respectively. Bars with different letters are significant according to Waller–Duncan multiple comparison test post hoc test at *p* < 0.05 using SPSS statistical analysis software.

The activity of studied EOs against the fungal isolates was illustrated in Figure 2. All studied EOs were able to inhibit the growth of the tested fungal isolates in a dose-dependent manner. In particular, the thyme EO showed the highest antifungal effect against all tested fungi even at the lowest concentration, except for *P. digitatum* where it showed the activity only at the higher concentrations (Figure 2). In addition, the thyme EO and cycloheximide showed the highest activity against *P. italicum* and *P. commune* (Figure 2). Regarding the anise, fennel and marjoram EOs, they showed the highest activity against *P. expansum* at the higher tested concentration (Figure 2). On the other hand, the fennel and marjoram EOs showed the lowest activity against *A. flavus*, even at the higher concentration (Figure 2).

### 3.3. Sensory Test

The evaluation of meat quality was done by humans using the scale of measurement as illustrated in Table 1. The advantage of the sensory test is to get a direct quantification of human perception of meat attributes insignificantly to the gender and age of the participants. The four major attributes of meat quality discussed in the current research are: color, odor, consistency and taste. In addition, the pH meter of the treated minced meat with each tested EO was measured and their values were ranged between 7.8 to 8.0, slightly different from the control sample (8.1). The overall results of the measured physical properties explicated that there is a general acceptance about the organoleptic parameters of meat after treatment with EOs through the evaluation of the participants.

**Figure 3:**
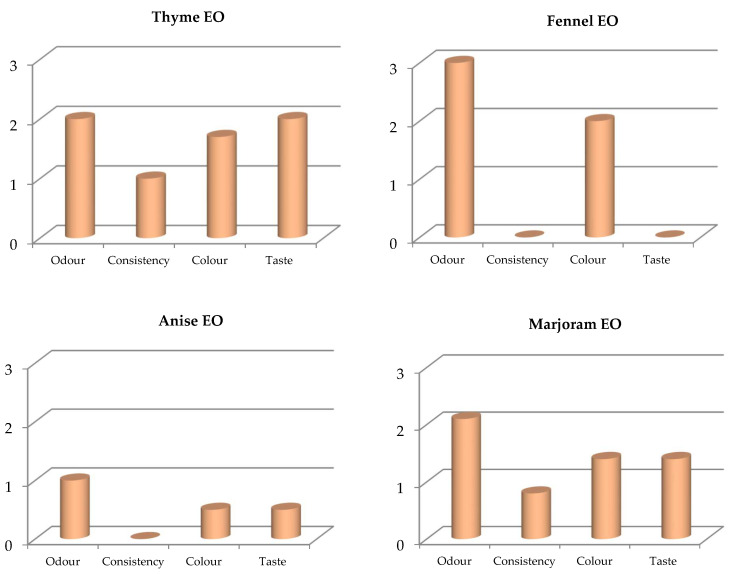
Sensory test of each single tested oil. Where the scale of the sensory test is: 0: No difference from control; 0–1: Similar to control; 1–2: low difference from the control; 2–3: highly different from the control. The used concentrations for the studied EOs were thyme 40 mg/mL; fennel 80 mg/mL; anise 60 mg/mL and marjoram 60 mg/mL.

**Figure 4:**
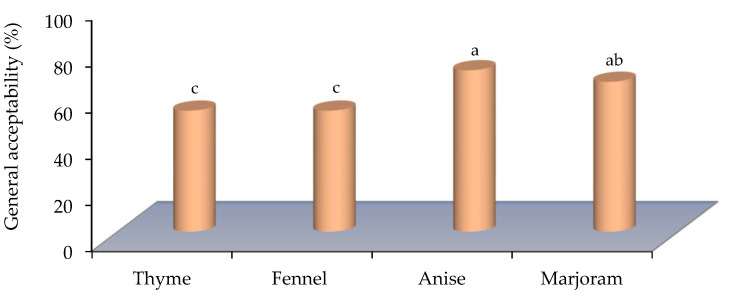
The consumer’s acceptability percentages of the studied essential oils. Where the percentage of general acceptability represents the total results of ten participants who selected the two lowest scale values of the organoleptic properties; (0) no difference from control, or (0–1) similar to control. Bars with different letters are significant according to Waller–Duncan multiple comparison test post hoc test at *p* < 0.05 using SPSS statistical analysis software.

In particular, meat treated with the thyme and marjoram EOs showed a similarity with the control (non-treated meat), especially with regard to consistency (Figure 3). However, meat treated with the fennel and anise EOs showed a great similarity with the control in terms of taste and consistency (Figure 3). In addition, the anise and marjoram EOs showed the highest significant value of the acceptability among the testers (Figure 4). Anyway, even the treated meat with the other EOs did not give a non-preferred odor and/or flavor as referred by the testers.

## 4. Discussion

The results of the current research have revealed the antimicrobial activity of the four studied EOs with different activity grades against the studied bacterial and fungal isolates from industrial meat products particularly consumed in the Middle East. The food-borne bacterial pathogens isolated from industrial meat products are the causal agents for important human diseases such as gastrointestinal infections, urinary tract infections, chronic opportunistic infections and some immunity system diseases [14,51]. Some fungal isolates from industrial meat products are able, under particular environmental conditions and substrates, to produce mycotoxins such as aflatoxins or ochratoxins [10,52,53,54] which have a serious toxic effect on human and animal health. In addition, the fungal species isolated in this study can also be a possible causal agents for human diseases such as pulmonary, skin mycosis, otomycosis, etc., [12,55].

The promising antimicrobial activity of several plant EOs which are usable, available and less dangerous for human health, shows that the EOs should be considered and undergoing some necessary examinations to control microbial contamination in food preservation, especially processed meat. The obtained results are in agreement with research that reported that some EOs such as *Schinus terebinthifolius* exerted efficient bactericidal effects against *Staphylococcus aureus*, *P. aeruginosa* and *E. coli* [56]. In addition, another study conducted by Bakkali et al. [57] reported that EOs extracted from *Verbena officinalis* L., *Majorana hortensis* L. and *Salvia officinalis* L. were able to inhibit the growth of some phytopathogenic bacteria in a dose-dependent manner such as *Clavibacter michiganensis*, *Bacillus megaterium* and *B. mojavensis*. In the case of antifungal activity, EOs of fennel, marjoram, oregano and sage exhibited a fungicidal effect against *B. cinerea* and *P. expansum* in apples [58]. Other studies reported that thyme and vervain EOs showed promising fungicidal effects higher than chemical preparations in postharvest treatments against *Monilinia laxa*, *M. fructigena* and *M. fructicola* on peach fruit [59].

As is widely known, the chemical composition of plant EOs is principally represented by mono- and sesquiterpene hydrocarbons and their oxygenated derivatives, along with aliphatic aldehydes, alcohols and esters [60].

In particular, monoterpenes are the most abundant compounds present in the four studied EOs. In fact, thyme oil was mainly characterized by the presence of high level of phenolic precursors and phenolic compounds such as *p*-cymene, *o*-cymene and carvacrol [46,61]. 

Regarding the marjoram EO, our previous research conducted by Elshafie et al. [49] revealed that its principal constituents are 1,8-cineole, α-pinene and limonene accounting for 33.5%, 9.0% and 6.4%, respectively. In addition, de Almeida et al. [62] reported that the main constituents of marjoram EO are (−)-citronellal (39.6%), carvacrol (13.3%) and *iso*-menthone (8.8%).

In the case of the fennel and anise EOs, our previous research conducted by Camele et al. [63] showed that they are composed mainly from cis-anethole 76.3 and 97.1, respectively, of the whole oil. In the same context, Tabanca et al. [64] reported that anise EO was constituted predominantly by E-anethole (94.2%), whereas Singh et al. [65] reported that fennel EO was mainly composed of anethole.

Obviously, the chemical composition of EOs is closely related to their antimicrobial effect [29]. Elshafie et al. [66] reported that the antimicrobial activities of several plant EOs depend mainly on their bioactive single components which are able to inhibit the growth of microorganisms and/or completely suppress the pathogens. In fact, according to another research, there may be a synergetic effect between two or more single chemical constituents that plays a distinctive function in the biological action of EOs [67]. The synergism between the aromatic plant components often plays an essential role in the effectiveness and reduction of the developing resistance of any pathogenic microorganism. Therefore, some constituents such as carvacrol, γ-terpinene and p-cymene are more effective when they are combined together [68]. On the other hand, Yousefi et al. [69] reported that there are various mechanisms of Eos’ antimicrobial action, such as increasing the cell permeability, changing the fatty acid profile of cell membranes and the inhibition of functional properties of the microbial cell wall. At the same time, antioxidant properties play an essential role in some of EOs biological activities, where the oxygenated compounds could play a role in the destruction of the microbial cell wall through increasing the cell permeability and finally lead to the complete cell death [29]. On the other hand, the differences in the antimicrobial activity from the four studied EOs could be related to the variation in their chemical composition. The monoterpenes are the most abundant compounds found in both thyme and marjoram EOs with 97.4% and 96.7, respectively, of the whole oil [63], whereas, the anise and fennel EOs are mainly constituted of nonterpenes representing 97.1 and 76.3%, respectively [63].

Regarding the sensory test, it is considered a significant survey in the production of foods, especially for processed meat products because they can provide useful information about their quality properties which represents the acceptability from the consumer’s side of the studied meat with the tested EOs. Many researchers have recently begun to investigate sensory testing as a means of producing health foods on the one hand, as well as supplying competitive marketplace products on the other. Through the evaluation of the participants, the overall findings of the measured organoleptic qualities revealed that there is a general acceptance of EOs-treated meat products and there is no significant difference to the control. On the other hand, it is mostly important to underline that during the cooking of meat the most part of EO volatile odour was eliminated, hence there was no notable odor by the testers.

## 5. Conclusions

The results of this study concluded that the studied plant EOs showed promising antimicrobial activity against some fungal or bacterial meat-contaminants. In particular, the thyme EO can be used effectively to control *P. fluorescence* and *E. coli* in processed meat products, whereas the marjoram EO could be used successfully for controlling *P. aeruginosa*. Furthermore, the thyme EO showed a promising activity against all tested fungal isolates, even at low concentration. However, the other tested EOs showed also reasonable results against some fungal isolates, particularly *Penicillium* species. In terms of the sensory test, the results revealed that the treatment of EOs did not significantly change the organoleptic properties of the meat product with respect to the control. Therefore, it is possible to use the tested EOs as potential natural meat preservatives as alternatives to synthetic chemicals. In fact, EOs can lower and/or completely inhibit the growth of several predominant foodborne-pathogens on one side and can also help to improve food safety and shelf life on another side without negative impact on the organoleptic properties of the processed meat products.

## Figures and Tables

**Table 1:** Scale of organoleptic properties of sensory test.

Scale	Definition
0	No difference from control
0–1	Similar to control
1–2	Low difference from control
2–3	Highly different from control

**Table 2:** Microbial total count in the minced meat products from five Egyptian regions.

	Samples	TBC (×10^5^)	*Pseudomonas* (×10^3^)	*Enterobacteriaceae* (×10^1^)	*E. coli*	Mould (×10^2^)	Yeast (×10^2^)
Minced meat	CA	6.4 ± 0.8 a	10 ± 7.5ab	6.2 ± 2.3 a	+	1.2 ± 0.6 a	1.8 ± 0.2 b
	GZ	54 ± 16 a	10.8 ± 2.3 ab	17.4 ± 10 a	++	1 ± 0.8 a	15 ± 7 a
	QA	78 ± 10 a	30 ± 12 a	79.8 ± 12 a	++	0.2 ± 0.1 a	4.6 ± 2.8 ab
	SH	9.33 ± 2 a	0.67 ± 0.2 b	3.33 ± 1.3 a	+	0.2 ± 0.1 a	1.3 ± 0.7 b
	BH	16 ± 1.3 a	18 ± 9 ab	2.8 ± 0.4 a	+	1.2 ± 0.3 a	2.2 ± 1.5 b

Values are represented as the average numbers of colonies per one gram of sample multiplied per the dilution factor as illustrated in each column (±SDs). In the case of *E. coli*, the results were represented as “+, ++” equal to “<35, 36–70 and >70%” of total samples of each region, respectively. Values followed by different letters in each vertical column were significantly different according to Waller–Duncan multiple comparison test post hoc test at *p* < 0.05.

**Table 3:** Microbial total count in the sausage product from five Egyptian regions.

	Samples	TBC (×10^5^)	*Pseudomonas* (×10^3^)	*Enterobacteriaceae* (×10^1^)	*E. coli*	Mould (×10^2^)	Yeast (×10^2^)
Sausage	CA	320 ± 94.9 b	24 ± 8.1 ab	118 ± 43.4 ab	+	1.6 ± 0.8 a	0.6 ± 0.5 c
	GZ	2 ± 2.2 c	18 ± 7.4 ab	14.8 ± 5.6 b	++	2.6 ± 1 a	2.2 ± 1.2 bc
	QA	42.5 ± 8 bc	57.5 ± 12.1 a	205 ± 81.5 a	++	0.75 ± 0.3 a	12.25 ± 3 a
	SH	850 ± 67.7 a	12 ± 1.0 c	22 ± 9 b	+	2 ± 0.6 a	6.5 ± 2 b
	BH	200 ± 42.4 ab	10 ± 2.4 c	2.6 ± 0.8 b	+	0.8 ± 0.2 a	2.2 ± 0.3 bc

Values are represented as the average numbers of colonies per one gram of sample multiplied per the dilution factor as illustrated in each column (± SDs). In the case of *E. coli*, the results were represented as “+, ++” equal to “< 35, 36–70 and >70%” of total samples of each region, respectively. Values followed by different letters in each vertical column were significantly different according to Waller–Duncan multiple comparison test post hoc test at *p* < 0.05.

**Table 4:** Microbial total counting in the burger product from the five Egyptian regions.

	Samples	TBC (×10^5^)	*Pseudomonas* (×10^3^)	*Enterobacteriaceae* (×10^1^)	*E. coli*	Mould (×10^2^)	Yeast (×10^2^)
Burger	CA	50 ± 4.6 ab	260 ± 51.6 a	158 ± 15 a	++	1.2 ± 0.9 a	1 ± 0.3 b
	GZ	4.4 ± 3.3 b	3.4 ± 4.2 b	53.6 ± 7 bc	+	2.4 ± 0.4 a	6.6 ± 2 ab
	QA	126 ± 67.5 a	100 ± 10 ab	16.6 ± 6 bc	++	0.47 ± 0.2 a	18.2 ± 8 a
	SH	3.8 ± 1.1 b	120 ± 65 ab	106 ± 20 ab	+	1.4 ± 0.5 a	2.8 ± 0.8 ab
	BH	28 ± 9.2 ab	12 ± 7 b	2.2 ± 2.2c	+	0.8 ± 0.2 a	1 ± 0.5 b

Values are represented as the average numbers of colonies per one gram of sample multiplied per the dilution factor as illustrated in each column (± SDs). In the case of *E. coli*, the results were represented as “+, ++” equal to “< 35, 3670 and >70%” of total samples of each region, respectively. Values followed by different letters in each vertical column were significantly different according to Waller–Duncan multiple comparison test post hoc test at *p* < 0.05.

## Data Availability

The data presented in this study are available on request from the corresponding author.
